# Irreversible Acquired Noncompaction Cardiomyopathy in a Parturient with Corrected Atrial Septal Defect: A Case Report and Clinical Implications

**DOI:** 10.1155/2020/1937589

**Published:** 2020-02-29

**Authors:** Is'haq Al-Aamri, Simone Derzi, Albert Moore, Natalie Bottega, Maria V. Ordoñez, Valerie Villeneuve, Roupen Hatzakorzian

**Affiliations:** ^1^Department of Anesthesiology, Royal Victoria Hospital-Glen Site, McGill University Health Center, Montréal, QC, Canada; ^2^Departments of Cardiology, Royal Victoria Hospital-Glen Site, McGill University Health Center, Montréal, QC, Canada; ^3^Critical Care Medicine, Royal Victoria Hospital-Glen Site, McGill University Health Center, Montréal, QC, Canada

## Abstract

Left ventricular noncompaction (LVNC) is described as a cardiomyopathy with an increase in left ventricle trabeculations and recesses. We report a rare case of persistent pregnancy-acquired LVNC cardiomyopathy and review the anesthetic peripartum management strategies. A 33-year-old parturient was followed closely by the high-risk obstetric service for her second pregnancy. She had an unresolved LVNC cardiomyopathy that was diagnosed during her first pregnancy for which she had a caesarean section. Her symptoms included occasional palpitations and dyspnea. She was started on metoprolol and enoxaparin. A successful caesarean section was performed at 37 weeks gestation under regional anesthesia. Echocardiograms prior to and during the second pregnancy demonstrated persistence of the LV hypertrabeculations, LV systolic dysfunction, and a left ventricular ejection fraction (LVEF) of 35%. Pregnancy-induced LV hypertrabeculations occur in a significant proportion of women, but most cases spontaneously resolve completely. Favorable maternal and fetal outcomes require multidisciplinary care and careful selection of the anesthetic technique and drugs that maintain stable hemodynamics.

## 1. Introduction

Left ventricular noncompaction (LVNC) is a rare cardiomyopathy characterized by an increase in left ventricle (LV) trabeculations and recesses. Both congenital and acquired cases have been reported [1]. Diagnosis of LVNC is based on morphological features of the LV and clinical presentation. Echocardiography has been used as the routine initial noninvasive diagnostic test to facilitate the identification and assessment of LVNC. It evaluates the presence of LV myocardial trabeculations and a 2-layer distinction between compacted and noncompacted myocardium [2]. Moreover, development of hypertrabeculations during pregnancy has been described, with most improving after delivery or resolving completely [3]. We report a case of unresolved pregnancy-acquired LVNC cardiomyopathy.

## 2. Case Presentation

We obtained written informed patient consent for this report. A 33-year-old parturient (gravida 2, para 1) with a history of congenital atrial septal defect (ASD) closure at the age of 10 years presented to our high-risk obstetric anesthesia service for consultation during her second pregnancy.

**Figure 1:**
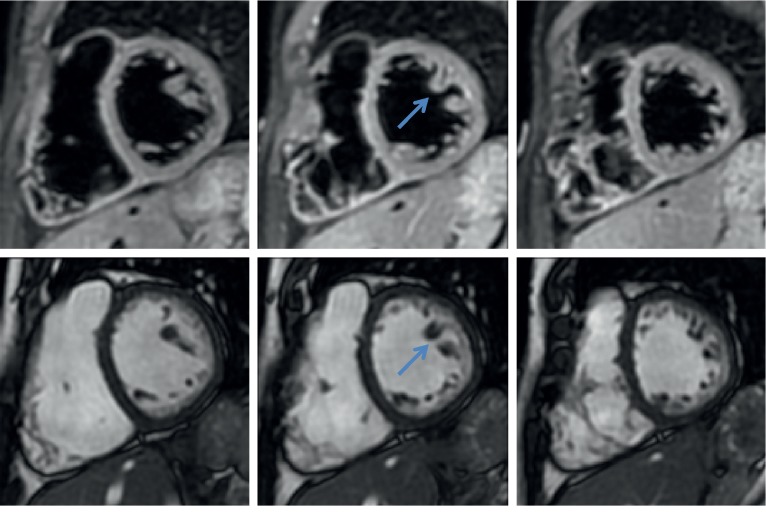
MRI 2 months after the first delivery demonstrated a ratio of the thicker noncompacted endocardial layer (N) and thin epicardial compacted layer (C) (N/C ratio) ≥2.0.

Three years earlier, the patient had presented with dyspnea during her first pregnancy. An echocardiogram demonstrated new LV hypertrabeculations and LVEF of 45%. Consequently, she was diagnosed with acquired LVNC cardiomyopathy. The patient was managed with furosemide and metoprolol. Vaginal delivery was unsuccessful for her first pregnancy as ventricular arrhythmias and congestive heart failure during labor necessitated an emergency caesarean section. Cardiac magnetic resonance (CMR) imaging and echocardiogram performed two months after the first delivery confirmed LVNC and demonstrated a ratio of the thicker noncompacted endocardial layer (N) and thin epicardial compacted layer (C) (N/C ratio) ≥2.0, thus meeting the criteria for LVNC (Figure 1). Furthermore, there was a late gadolinium enhancement on CMR in the infero-lateral wall of the midventricle and apex.

**Figure 2:**
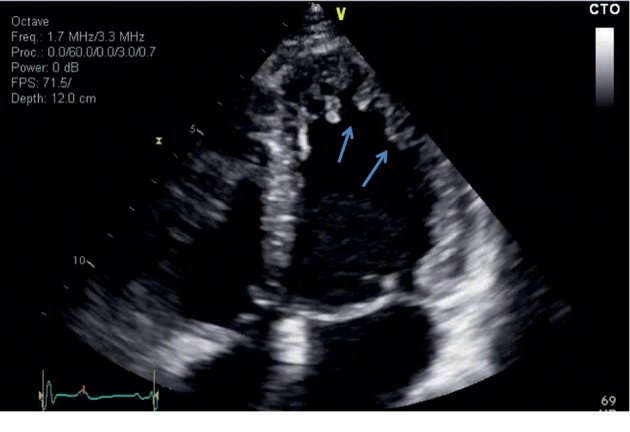
Three months after the second delivery: transthoracic echocardiographic apical 4-chamber view showed hypertrabeculation in the LV apex and lateral wall.

Throughout her second pregnancy, she was followed closely by a multidisciplinary team including cardiology, high-risk obstetrics, and anesthesiology. During the second trimester, she was started on metoprolol 12.5 mg twice daily and enoxaparin 40 mg subcutaneously once daily. At this time, her main symptoms comprised occasional palpitations and shortness of breath (New York Heart Association II). A 24 hr Holter monitor was requested in the 3^rd^ trimester and postpartum which showed some paroxysmal ventricular contractions with no fatal arrhythmias. She was booked for an elective caesarean section at 37 weeks of gestation. In the operating room, the standard American Society of Anesthesiologists monitors were placed on the patient. A right radial arterial line and two large-bore intravenous catheters were placed. Titrated epidural anesthesia was done with 2% lidocaine over 15 min. Adequate surgical anesthesia was achieved with 20 ml of 2% lidocaine. Patient remained hemodynamically stable throughout the caesarean section. Postoperatively, she had an uncomplicated postpartum recovery in the intensive care unit and was discharged home 5 days after delivery. Echocardiograms prior to, during, and after the second pregnancy demonstrated persistence of the LV hypertrabeculations, LV systolic dysfunction, and an LVEF of 35% (Figure 2).

## 3. Discussion

LVNC is a rare cardiomyopathy, which is characterized by a two-layered structure of the myocardium consisting of a compacted, thin epicardial layer and a noncompacted, severely thickened endocardial layer. Traditionally, LVNC is diagnosed by echocardiography. The criteria by Chin et al. and Jenni et al. to evaluate the presence of LVNC are commonly used. These criteria include a bilayered myocardium, a noncompacted-to-compacted ratio >2 : 1, communication with the intertrabecular space demonstrated by Doppler, and presence of multiple prominent trabeculations in the end systole [4, 5]. Cardiac magnetic resonance has allowed improvement in differentiating the noncompacted and compacted myocardium. An important characteristic of CMR in addition to the spatial resolution is the ability to image the apex well. Also, late gadolinium enhancement imaging can be obtained which is a surrogate of myocardial fibrosis. There appears to be a correlation of fibrosis with the clinical severity and the ventricular dysfunction in patients with noncompaction disease [6]. The prevalence of LVNC in adults ranges from 0.14%–0.26% in patients referred for echocardiography and 8–33% in patients with a history of familiar cardiomyopathy [1, 2]. Clinical presentation is variable and may be asymptomatic (17%), overt heart failure (47%), arrhythmias (11%), and/or thromboembolic events (10%) [2]. Asymptomatic *de novo* LV trabeculations in pregnancy have been reported in 25% of pregnant women, where 73% had complete resolution of trabeculations over the next 24 months [3]. Hypertrabeculation of the LV is hypothesized to be mediated by the increase in preload during the third trimester; this phenomenon has been observed in patients with sickle cell anemia and in athletes [7]. However, the development and progression of LVNC in pregnancy is not well defined, and current literature is limited to case reports. There were no hypertrabeculations documented echographically in our patient until dyspnea developed at 30 weeks into her first pregnancy, which is consistent with the current description in the literature.

An LVNC cardiomyopathy parturient should be delivered in a tertiary care center where the potential of acute cardiac decompensation during surgery can be managed. Preterm delivery may become necessary if rapid deterioration of LV function occurs. Involvement of the anesthesia team should be done early in the beginning of the third trimester and immediately if the patient presents in labor. Anesthesia management of LVNC cardiomyopathy during labor is limited in the literature to a few case reports. Most case reports showed severe presentation during pregnancy which could be a bias in managing patients with compensated disease. However, uneventful vaginal delivery and caesarean section has been reported [8]. Early labor epidural is recommended to reduce sympathetic response caused by painful contractions and as well to enhance reduction in afterload. However, the neuraxial technique can be contraindicated in patients on anticoagulation presenting in active labor or emergency caesarean section. Vaginal delivery is an option for patients with asymptomatic or compensated cardiomyopathy. Forceps or vacuum-assisted delivery will reduce exertion and shorten the second stage of labor. In severe cases, elective caesarean section should be considered with invasive monitors. Neuraxial anesthesia induces a sympathectomy-related decrease in systemic vascular resistance (SVR). In our patient, a slow titrated epidural was used rather than combined or spinal anesthesia during caesarean section to minimise any hemodynamic changes. General anesthesia is used in cases where there is a severe compromise of left ventricular function with or without pulmonary hypertension as this allows an easier management of afterload reduction and cardiac output [9].

Koster et al. reported the use of extracorporeal membrane oxygenator (ECMO) as a standby in a patient with severe LVNC cardiomyopathy and pulmonary hypertension [10]. Postoperatively, patients should be admitted to a monitored setting. Gradual recovery of neuraxial block and autotransfusion from the contracting uterus can worsen the heart failure, so we recommend to keep the epidural catheter for a few hours and use furosemide early in postpartum as needed.

Noncompaction cardiomyopathy that develops in a parturient may persist to impact future pregnancies. There is limited data on the prognosis and on the probability of relapse of pregnancy-acquired LVNC. Most studies examining peripartum cardiomyopathy have demonstrated recovery in LV function in 58–68% of women, with heart failure or relapse in 15–17% in the subsequent pregnancy, mostly in parturients with unrecovered LV function [11]. In our case, the hypertrabeculations persisted into the second pregnancy with lower LVEF (35%).

The management of LVNC cardiomyopathy is based on treating heart failure and on arrhythmia prevention. In addition, these patients have a higher risk of thromboembolic events due to pregnancy-induced hypercoagulable state. Beta-blockers are well tolerated during pregnancy, although they are associated with a higher incidence of fetal growth restriction, particularly with atenolol. Digoxin can also be considered in systolic heart failure during pregnancy [12]. Our patient was managed with diuretics (furosemide) and beta-blockers (metoprolol) when she presented with heart failure in the first pregnancy. Once her symptoms improved, the diuretic was stopped and the beta-blocker maintained. For her second pregnancy, treatment involved metoprolol again as well as enoxaparin. Fetal ultrasound in her second pregnancy showed normal growth.

The prevention of thromboembolic complications in LVNC is uncertain. Our patient was started on prophylactic anticoagulation with low molecular weight heparin (LMWH). Although LMWH reduces the risk of deep venous thrombosis, there is no evidence for the prevention of arterial thrombus formation, nor is there evidence for clot prevention in LVNC cardiomyopathy. Oechslin et al. showed significant incidents of thromboembolic events during long-term follow-up of patients with isolated ventricular noncompaction; they recommend oral anticoagulation for every patient in whom LVNC is diagnosed [13]. Murphy et al. recommended anticoagulation to patients with impaired systolic function (LVEF < 25%), a history of thromboembolism, or a history of transient ischemic attack with normal LV function or dense and extensive apical LVNC [14].

Moreover, in a pediatric review by Stolberger et al., 64% of LVNC was associated with congenital cardiac anomalies, with 51% of LVNC associated with ASD [15]. Of note, our patient's prior history of ASD may suggest an association between congenital heart disease and the risk of developing LV hypertrabeculations in pregnancy, an association that needs further evaluation.

Patients with LVNC are at increased risk of life-threatening ventricular arrhythmias and sudden cardiac death. Kobza et al. concluded that implantable cardioverter-defibrillators (ICD) are effective for the primary or secondary prevention of sudden cardiac death in patients with LVNC [16]. The Canadian Cardiovascular Society recommends ICD insertion in all patients who have persistent low LV function (LVEF < 30%) after three months of oral medical therapy for the management of heart failure [17]. As our patient had persistent low LV function (LVEF 35–40%) but no sustained ventricular tachycardia, the decision was made not to insert an ICD. Finally, echocardiographic screening is recommended in first-degree relatives of patients with LVNC. In our case, the parturient had no siblings, and she was made aware that her children should be screened and followed closely for LVNC.

## 4. Conclusion

Pregnancy-induced LVNC cardiomyopathy can present as heart failure, severe arrhythmias, or thromboembolism. During pregnancy, regular follow-up and assessment of LV function is indicated. The management is directed toward the treatment of heart failure and preventing complication such as arrhythmias and thromboembolic events. ICD and anticoagulation should be considered in patients with persistently low LVEF. Favorable maternal and fetal outcomes require multidisciplinary care and careful selection of the anesthetic technique and drugs that maintain stable hemodynamics.
